# Does Short-Term Exposure to Mobile Phone Base Station Signals Increase Symptoms in Individuals Who Report Sensitivity to Electromagnetic Fields? A Double-Blind Randomized Provocation Study

**DOI:** 10.1289/ehp.10286

**Published:** 2007-07-25

**Authors:** Stacy Eltiti, Denise Wallace, Anna Ridgewell, Konstantina Zougkou, Riccardo Russo, Francisco Sepulveda, Dariush Mirshekar-Syahkal, Paul Rasor, Roger Deeble, Elaine Fox

**Affiliations:** University of Essex, Colchester, Essex, United Kingdom

**Keywords:** base station, electromagnetic fields, electromagnetic hypersensitivity, mobile phone, well-being

## Abstract

**Background:**

Individuals with idiopathic environmental illness with attribution to electromagnetic fields (IEI-EMF) believe they suffer negative health effects when exposed to electromagnetic fields from everyday objects such as mobile phone base stations.

**Objectives:**

This study used both open provocation and double-blind tests to determine if sensitive and control individuals experience more negative health effects when exposed to base station-like signals compared with sham.

**Methods:**

Fifty-six self-reported sensitive and 120 control participants were tested in an open provocation test. Of these, 12 sensitive and 6 controls withdrew after the first session. The remainder completed a series of double-blind tests. Subjective measures of well-being and symptoms as well as physiological measures of blood volume pulse, heart rate, and skin conductance were obtained.

**Results:**

During the open provocation, sensitive individuals reported lower levels of well-being in both the global system for mobile communication (GSM) and universal mobile telecommunications system (UMTS) compared with sham exposure, whereas controls reported more symptoms during the UMTS exposure. During double-blind tests the GSM signal did not have any effect on either group. Sensitive participants did report elevated levels of arousal during the UMTS condition, whereas the number or severity of symptoms experienced did not increase. Physiological measures did not differ across the three exposure conditions for either group.

**Conclusions:**

Short-term exposure to a typical GSM base station-like signal did not affect well-being or physiological functions in sensitive or control individuals. Sensitive individuals reported elevated levels of arousal when exposed to a UMTS signal. Further analysis, however, indicated that this difference was likely to be due to the effect of order of exposure rather than the exposure itself.

Radio frequency electromagnetic fields (rf-emf) do not fall within the ionizing spectrum. Nevertheless, high-intensity rf-emf can cause thermal effects with serious implications for human health (Conway 2001). In everyday life, however, most humans are not exposed to such high intensity rf-emf and do not possess sensory organs that can detect electric or magnetic fields. The question remains as to whether exposure to low-intensity rf-emf, even if undetected, can negatively affect human health. A subgroup of the population has claimed that they are sensitive to rf-emf and this condition, formerly known as electromagnetic hypersensitivity, has recently been relabeled in a World Health Organization workshop (Hansson Mild et al. 2006) as idiopathic environmental intolerance with attribution to electromagnetic fields (IEI-EMF). In a recent U.K. survey, it was reported that around 4% of people claim that they are sensitive to rf-emf to some degree (Eltiti et al. 2007). A variety of negative health effects (e.g., cold and flu-like symptoms) are attributed to exposure to rf-emf from objects such as computers and mobile phones. Previous research has indicated that IEI-EMF individuals report lower levels of well-being compared with healthy individuals (e.g., Eltiti et al. 2007; Regel et al. 2006; Rubin et al. 2005; Zwamborn et al. 2003) and that the symptoms they experience may greatly affect their quality of life (e.g., Bergqvist and Vogel 1997; Irvine 2005). However, evidence that IEI-EMF symptoms are indeed caused by rf-emf exposure is yet to be established. A systematic review of 31 blind and double-blind provocation studies yielded no evidence that IEI-EMF individuals could detect the presence of rf-emf, and only seven studies indicated that exposure to rf-emf did affect health indices (Rubin et al. 2005). In two of these studies, however, the authors failed to replicate their own findings. Another four studies involved inappropriate use of statistics, while one reported improved mood in the active exposure condition. One unpublished double-blind study that specifically examined base station signals did find that exposure to a universal mobile telecommunications system (UMTS) signal resulted in reduced subjective well-being for both sensitive and nonsensitive individuals, whereas a global system for mobile communication (GSM) base station signal had no effect (Zwamborn et al. 2003). However, a recent study conducted in Switzerland was unable to replicate this effect (Regel et al. 2006). Another double-blind study recently reported no negative health effects from exposure to a standard 900 MHz GSM handset signal for either sensitive or control participants (Rubin et al. 2006).

The existing evidence therefore indicates that exposure to rf-emf signals from mobile phone base stations and handsets has little effect on health, even in those individuals with a perceived sensitivity to rf-emf. Nevertheless, only two double-blind studies have been conducted with base station signals, with contrary results. Given the increase in mobile phone base stations around the world and the level of public concern regarding possible negative health implications, further research is necessary to investigate the short-and long-term impact of exposure to rf-emf in both healthy and IEI-EMF groups.

The current study tested whether short-term exposure to typical GSM and UMTS base station signals affected a variety of measures of well-being in sensitive and control individuals, using both open provocation and double-blind tests. It was hypothesized that sensitive participants would report more symptoms and lower levels of well-being during GSM and UMTS exposures compared with sham. In addition, sensitive participants should be able to identify above chance level whether the base station was turned “on” or “off.” For control participants no difference was expected in the number or severity of symptoms reported during exposures. Previous research has reported higher levels of heart rate, heart rate spectrum ratio, and electrodermal activity in sensitive compared with control individuals (e.g., Lyskov et al. 2001a, 2001b). Thus, physiological measurements were also conducted to determine whether exposure to GSM and UMTS base station signals affected objective measures of well-being in both sensitive and control individuals.

## Methods

### Participants

**Figure 1:**
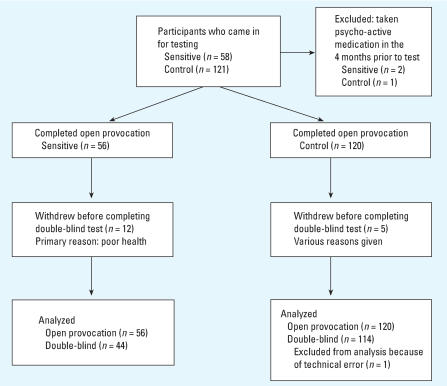
Flow chart of sensitive and control participation in open provocation and double-blind tests.

Fifty-eight self-reported sensitive and 121 control individuals presented for testing. Of these, 56 sensitive and 120 controls completed the open provocation test, while 44 sensitive and 115 controls also completed the double-blind tests (see Figure 1 for flow diagram of participation). Before testing, all participants completed the Electromagnetic Hypersensitivity Questionnaire (Eltiti et al. 2007), which allowed the researchers to assess their current state of health and whether the individual attributed their symptoms to exposure to rf-emf. Participants in the sensitive group self-reported experiencing negative health effects from electromagnetic field exposure, particularly exposure from mobile phones and/or mobile phone base stations, whereas those in the control group did not report experiencing any negative health effects from rf-emf exposure. Individuals who had suffered a brain injury, were currently suffering from epilepsy or claustrophobia, had been fitted with pacemakers, had previously undergone treatment for a mental disease, or had taken psycho-active medication in the 4 months prior to testing were excluded from participation. Participants were recruited through local advertising, action groups, and word of mouth. In addition, some participants had previously participated in a questionnaire study conducted by the research group (Eltiti et al. 2007).

All testing was conducted at the Electromagnetics and Health Laboratory at the University of Essex, Colchester, United Kingdom. Participants were reimbursed for their travel expenses and received a small payment for participation. The study was approved by the University of Essex ethics committee. All participants gave written informed consent before proceeding with testing.

### Design

The study was a mixed design in which participants were exposed to three conditions: GSM, UMTS, and sham. Each participant took part in four testing sessions, which occurred at least 1 week apart at approximately the same time of day (± 3 hr). Session 1 consisted of an open provocation and a quick double-blind test. During the open provocation both the participants and experimenters knew when the base station was “on” and “off” and, if it was “on,” whether it was emitting a GSM or UMTS signal. During the double-blind tests neither the participants nor experimenters knew which exposure was being generated. Sessions 2, 3, and 4 each consisted of a single exposure condition (GSM, UMTS, or sham) and these were double-blind. Counterbalancing of all the exposures was preprogrammed into the exposure system control computer, for a target of 264 participants (132 sensitive and 132 controls). Assuming there is a small effect of rf-emf on human health (*d* = 0.40) and that sometimes this effect is positive and sometimes negative (two–tailed), it was calculated that 66 participants per group were needed to have a power level of 0.90 to detect a within-subjects effect (i.e., difference between real and sham exposure conditions) and 132 participants per group were needed to detect a between-subjects effect (i.e., group by exposure condition interaction), for a total of 264 participants (Howell 1997). For each test the researcher simply entered the participant and session number into the computer, and the preprogrammed exposure condition was generated. Thus, the study consisted of three types of exposure (GSM, UMTS, sham) and two groups (sensitive and control). The dependent variables were various measures of subjective well-being and physiological functioning.

### Materials and equipment

#### Screened room

All testing took place in the Electromagnetics and Health Laboratory, which comprised a testing room, reception area, and experimenter’s room. The testing room was 7 m × 4 m × 2.4 m and had a shielding effectiveness greater than 60 dB at the tested frequency range. Participants were seated exactly 5 m from the base station antenna, which was blocked from view by a screen (2.8 m from the participant) upon which instructions were projected. The projector was located outside the testing room, with projection made through a screened window located on the wall behind the antenna. A screened window (47 cm × 47 cm) on the near wall enabled constant visual contact between the participant and experimenter.

#### Exposure system

There were three exposure conditions: GSM, UMTS, and sham. Both the GSM and UMTS exposures were designed to propagate a signal that replicated as closely as possible those generated by actual base stations in the environment. The GSM signal was a combined signal of both 900- and 1,800-MHz frequency bands, each with a power flux density of 5 mW/m^2^, resulting in a combined power flux density of 10 mW/m^2^ over the area in which the participant was seated. The GSM signal contained both broadcast channels (886.8 and 1,877 MHz) and traffic channels (888.8 and 1,879 MHz). The eight time slots on the broadcast channels were always occupied, while changes in the power level of the traffic channels were simulated using two first-order, two-state Markov processes, assuming a blockage rate of 1% and call activity of 40%. This provided a realistic approach for traffic channel modeling similar to that carried by live base stations during peak hours, resulting in the traffic channels having a blockage rate of 1% and a call activity of 40%. The time slot occupancy of the GSM signal consisted of eight time slots, each with a duration of 576.875 μsec, resulting in a total frame duration of 4.615 msec. Interslot guard intervals of 32 μsec duration were implemented into each GSM frame, with a drop in power level of around 50 dB between the active state (the burst) and the inactive state (the guard).

The UMTS signal had a frequency of 2,020 MHz with a power flux density of 10 mW/m^2^ over the area where the participant was seated. Traffic modelling for the UMTS signal was achieved using test model 1, as defined by the 3rd Generation Partnership Project standard. This model represented a realistic traffic scenario, with high peak to average ratio power changes, and also ensured both repeatability and parameter control over the UMTS exposure.

During the sham condition the power level was nil and no signal was transmitted. The stability of the exposure system was checked and calibrated every 6 months and was found not to exceed ± 3 dB of tolerance at any of the three frequency bands. All base station signals and field uniformity were independently tested and verified by the National Physical Laboratory.

Signals were generated using a Rohde and Schwarz SMU200, which was connected to a diplexer, an interslot trigger module, a power amplifier, a through-line power meter, a controller personal computer (PC), and an antenna. The diplexer enabled the mixing of the 900- and 1,800-MHz signals to create the GSM exposure, whereas the power amplifier enabled the signal to be set at the correct power level. The through-line power meter was used to perform continuous checks of the power into the antenna during the tests. The operator was informed if the power level exceeded the tolerance value. The controller PC regulated all the exposures, giving the system both repeatability and full control over the parameters for each exposure. A copy of the technical reference manual is available upon request (Belloul 2006).

#### Subjective well-being

Subjective well-being was measured using visual analogue scales (VAS) and symptom scales. The VAS consisted of 10-cm lines anchored at one end with the phrase “not at all,” at the other with “extremely” and measured anxiety, tension, arousal, relaxation, discomfort, and fatigue. The corresponding words used to anchor the lines were “anxious,” “tense,” “agitated,” “relaxed,” “discomfort,” and “tired.” The symptom scales consisted of a list of 57 symptoms extracted from the Electromagnetic Hypersensitivity Questionnaire (Eltiti et al. 2007) in which participants indicated how much they were suffering from each symptom, from “not at all” to “a great deal.”

#### Physiological measures

The dependent variables for blood volume pulse (BVP), skin conductance (SC), and heart rate (HR) were the mean (M) and standard deviation (SD) values calculated for the 15-min open provocation and 50-min double-blind tests. The physiological measurements of BVP, HR, and SC were recorded using a ProComp Infiniti 8 channel encoder with Biograph Infiniti software (Thought Technology Ltd., Plattsburgh, NY, USA; http://www.thoughttechnology.com/index.htm) run on a Dell Latitude notebook (Dell Products UK, Dublin, Ireland). Signals were sampled at a rate of 2,048 samples/sec for BVP and 256 samples/sec for SC. The BVP was submitted to a 4th order Butterworth low-pass filter with a 10-Hz cutoff frequency. The HR was calculated from the filtered BVP by calculating the time locations for the BVP peaks and valleys on the basis of the locations on which the derivative of the BVP reached zero (dicrotic notches were discarded). HR was then estimated on the basis of the time between peaks: HR = 1/(interpeak interval). All signals were resampled at eight samples/sec in order to have a uniform rate. BVP signals were detrended, as the important information in this signal was on the peak-to-peak values.

#### “On”/”off” judgments

For the three quick double-blind tests (in session 1) and the three 50-min double-blind tests (sessions 2–4), participants judged whether the base station was “on” or “off” and indicated how confident they were of this judgment using a scale from 0 “not at all sure” to 100 “completely sure.” We chose the receiver operating characteristic (ROC) curve method to analyze the responses, as this takes into account not only accurate (hits) and inaccurate responses (false alarms) but also how confident participants are of their judgments.

#### Procedure

Testing took place on four separate occasions at least 1 week apart, with one participant tested at a time. During session 1, informed consent and background information, including a medical history, were taken, and the cognitive tests (to be reported elsewhere), open provocation, and quick double-blind tests were performed. During the open provocation and quick double-blind tests, participants received all three exposures. Sessions 2, 3, and 4 each consisted of a single exposure (GSM, UMTS, or sham) and were all double-blind, with the three exposures being randomly spread across the three sessions. Session 1 took approximately 3 hr to complete, whereas sessions 2, 3, and 4 each took approximately 1.5 hr. Full details of the sessions are listed in Table 1.

## Results

### Exposure

As we were unable to reach our target of 264 participants, we could not guarantee complete counterbalancing of order of exposures across sessions for the double-blind tests. Chi-square analysis revealed that there were no significant differences between the groups and order of exposure for the double-blind tests; however, almost half the sensitive group received the UMTS exposure first (45.5%) compared with the GSM first (27.3%) or sham first (27.3%). The order of exposure was more evenly distributed for the control group, with 35.1% receiving sham first, 36.0% receiving GSM first, and 28.9% receiving UMTS first.

### Biographical information

The sensitive group (M = 46.1, SD = 13.5) was significantly younger than the control group [M = 54.5, SD = 15.23; *t*-test (*t*) (174) = −3.51, *P* < 0.01], with equal numbers of males and females in each group (sensitive: male 57.1%; control: male 57.5%; χ^2^ (1) = 0.002, *P* > 0.05). Significantly more controls (38.3%) reported having a chronic illness compared with sensitive participants [21.4% ; χ^2^ (1) = 4.94, *P* < 0.05], although there were no differences between the groups among the five most commonly reported chronic illnesses: high blood pressure, underactive thyroid, high cholesterol, asthma, and arthritis (χ^2^’s (1) < 4.5, *P*’s > 0.01). Bonferroni corrections were applied to all multiple comparisons to reduce the likelihood of familywise alpha errors.

### Visual analog scales

The data for the VAS were skewed, mainly due to individuals reporting close to the end points. The data were therefore transformed into normal distributions using the square root transformation. The relaxation VAS was reversed from the others; therefore, it was transformed using the reflect and square root transformation [SQRT(10-*X*)]. A 3 (condition: sham, GSM, UMTS) X 2 (group: sensitive, control) mixed analysis of variance (ANOVA) was performed on the transformed data for each VAS separately for the open provocation and double-blind tests (see Table 2 for means, standard errors, and *F*-and *t-*test values). For the open provocation, all VAS resulted in a significant main effect for group, with sensitive participants reporting higher levels of anxiety, tension, arousal, discomfort, and fatigue than controls, whereas controls reported higher levels of relaxation than sensitive participants. The main effect for condition [*F*’s (2,346) > 10.04, *p’*s < 0.001] and the interaction between condition and group was significant for all VAS except fatigue. Paired sample *t*-tests showed a significant difference between sham and GSM and between sham and UMTS conditions for sensitive participants but not for controls. Sensitive individuals reported higher levels of anxiety, tension, arousal, and discomfort and lower levels of relaxation during the GSM and UMTS conditions compared with the sham condition.

The results for the double-blind data were similar, with a significant main effect of group for all VAS and of condition for anxiety, tension, and arousal [*F*’s(2,312) > 3.00, *p*’s ≤ 0.05]. Of more interest, there were significant conditions by group interactions for anxiety, tension, arousal, and relaxation. Paired samples *t*-tests revealed higher levels of arousal during the UMTS compared with sham condition for the sensitive group only, as shown in Table 2. A problem in interpreting this significant effect is that a larger proportion of sensitive individuals received the UMTS compared with GSM or sham exposure in session 2 (the first of the 50-min double-blind conditions). Examination of the data showed that regardless of exposure condition, sensitive participants had a significantly higher degree of arousal during session 2 (M = 3.03) compared with session 3 [M = 2.34; *t*(43) = 2.64, *p* < .025], whereas there was no difference between sessions 3 and 4 [M = 2.32; *t*(43) = 0.47, *p* > 0.05]. To further test if there was a significant effect of the UMTS exposure on arousal when order of exposure was held constant, separate 2 (condition: UMTS, sham) X 2 (group: sensitive, control) between-subjects ANOVAs were performed for each session (see Table 3 for mean, standard error, and *F* values). The main effect for condition and group by condition interaction was not significant for all three sessions. These results indicate that the apparent increase in arousal with UMTS exposure was attributable to the higher proportion of sensitive individuals who received UMTS in session 2 (45.5%). It is important to note that regardless of type of exposure or session, all the VAS scores fell within the lower “not at all” end of the scale.

### Symptom scales

The majority of control individuals reported experiencing no symptoms in any condition; therefore, Wilcoxon signed-ranks tests were performed on the total number of symptoms reported and total symptom scores (see Table 4 for medians and Z scores). During the open provocation the sensitive group reported more symptoms and a higher total symptom score during the GSM and UMTS conditions compared with sham. The control group reported more symptoms during the UMTS compared with sham, but not for GSM compared with sham. During the double-blind tests there was no difference between active and sham exposures in either the total number of symptoms or the total symptom score for either group. Sensitive participants reported more symptoms than did controls, but this was not related to exposure condition.

### Physiological measures

Inspection of the physiological data revealed that it was skewed for all measurements except the HR (M). Square root transformations were applied to the BVP (SD), SC (M), and SC (SD). A logarithmic transformation was applied to the HR (SD) to form normal distributions (see Table 5 means, standard errors, and *F* values). The BVP (M) did not lend itself to transformation or analysis because of low kurtosis values. The data were analyzed using a 3 (condition: sham, GSM, UMTS) X 2 (group: sensitive, control) mixed ANOVA for the open provocation and double-blind tests. There was no difference between active and sham conditions regardless of type or even knowledge of exposure for either group. There was, however, a significant between-group difference in SC, with sensitive participants having higher SC (M and SD) responses during the open provocation and double-blind tests. The HR (SD) was also significantly higher in the sensitive group compared with that in the control group during the open provocation test. No other comparisons were significant.

### “On”/”off” judgments

**Figure 2:**
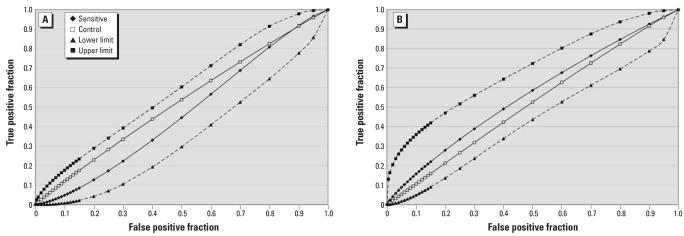
ROC curve and 95% CI values for “on”/”off” judgments for sensitive and control participants. (*A*) ROC curve for the 5-min double-blind sessions. (*B*) ROC curve for the 50-min double-blind sessions.

Participants made “on”/”off” judgments during both the 5-min and 50-min double-blind exposures. Sensitive participants had an accuracy rate of 55.2% during the 5-min tests (*d*′ = −0.08, sensitivity = 66.4%, specificity = 32.7%) and 59.8% during the 50-min tests (*d*′ = 0.20, sensitivity = 69.3%, specificity = 40.9%). The control group had an accuracy rate of 51.4% during the 5-min tests (*d*′ = 0.10, sensitivity = 51.7%, specificity = 50.8%) and 50.1% during the 50-min tests (*d*′ = 0.06, sensitivity = 48.0%, specificity = 54.3%). See Figure 2 for ROC curves and 95% confidence intervals (CI). For each group the 95% CI on the ROC curves includes the diagonal axis, implying that participant performance for each group did not differ from chance. Only two sensitive and five control participants were able to correctly identify all six “on”/ “off” judgments, while no one correctly distinguished between the GSM and UMTS signal 100% of the time.

## Discussion

Elevated levels of arousal were found under double-blind conditions for the sensitive participants during the UMTS compared with sham exposure, similar to the findings of Zwamborn et al. (2003). Further analysis revealed that this increased arousal was most likely due to a higher proportion of sensitive individuals receiving the UMTS signal first. It is not surprising that sensitive individuals would be more anxious in the first of the double-blind sessions, given the degree of uncertainty they may have felt in not knowing how the signal would affect them. This was reflected in the significant condition by group interaction for the anxiety-related measures of anxiety, tension, arousal, and relaxation. However, during sessions 3 and 4 the sensitive individuals knew what to expect and were overall less anxious. In addition, the elevated level of arousal was not reflected in either the number or severity of symptoms reported, or the intensity of physiological measurements. Control individuals did not report any differences in levels of well-being for the UMTS signal, which is consistent with the findings of Regel et al. (2006), and the GSM signal did not affect levels of well-being for either group.

The open provocation test verified that when sensitive individuals knew the base station was emitting either a GSM or UMTS signal, they self-reported lower levels of well-being and more symptoms than during the sham condition. This demonstrated that the laboratory conditions did not prevent sensitive individuals from reacting to either the GSM or UMS signals. In addition, the questionnaires and statistical analysis used to measure well-being and symptom severity were sensitive enough to detect these differences. Importantly, when these same exposures were presented under double-blind conditions in which order of exposure was considered, no differences were observed.

Consistent with previous research, sensitive individuals reported more symptoms and greater severity of symptoms and also displayed higher levels of SC than control individuals regardless of type of exposure (e.g., Regel et al. 2006; Rubin et al. 2006; Lyskov et al. 2001b). This elevated level of SC in IEI-EMF compared with control individuals may reflect either a psychophysiological stress response to participating in the study or a more general imbalance in autonomic nervous system regulation as suggested by Lyskov and colleagues (2001a). Further research in this area is needed to determine the physiological parameters in sensitive individuals that are significantly elevated compared those in with control individuals and if regulation of these parameters can help alleviate IEI-EMF symptoms.

The present data, along with current scientific evidence, led to the conclusion that short-term rf-emf exposure from mobile phone technology is not related to levels of well-being or physical symptoms in IEI-EMF individuals. Furthermore, IEI-EMF individuals are unable to detect the presence of rf-emf under double-blind conditions. It remains, however, that IEI-EMF individuals present with a range of distressing and serious symptoms and often have a very poor quality of life. Given the current findings, together with findings of related research (Rubin et al. 2005), it is imperative to determine what factors other than low-level rf-emf exposure could be possible causes of the symptoms suffered by IEI-EMF individuals, so that appropriate treatment strategies can be developed.

## Figures and Tables

**Table 1:** Procedures for open provocation and double-blind tests.

Session	Task	Duration	Wash-out period
Session 1			
Open provocation (e.g., sham, GSM, UMTS)	VAS completed every 5 min; symptoms reported; physiological measurements taken continuously	15 min for each exposure	2 min between each exposure
Cognitive tests^a^	Participants completed digit symbol substitution task and digit span task	8 min	
Quick double-blind test (e.g., GSM, UMTS, sham)	Participants made a judgment as to whether base station was “on” or “off” and how confident of this judgment they were using a scale from 0 (not at all sure) to 100 (completely sure). If participants thought base station was“ on,” they also indicated whether they believed it was the GSM or UMTS signal and how confident they were of this judgment from 0 to 100	5 min for each exposure	2 min between exposures
Sessions 2, 3, 4 double-blind (e.g., session 2: UMTS; session 3: GSM; session 4: sham)			
Low load	Participants watched “Blue Planet” video (BBC Worldwide Ltd. 2003) completed VAS every 5 min, and recorded any symptoms. Physiological measurements were taken continuously during the session	20 min	
High load	Participants performed mental arithmetic (e.g., adding and subtracting 2-digit numbers). Task interrupted every 5 min for participants to complete VAS and record any symptoms	20 min	
Cognitive tests	Participants completed digit symbol substitution task and digit span task	8 min	
“On”/”off” judgment	Same as in session 1, participants made a judgment as to whether the base station was “on” or “off”		

a The results of cognitive tests will be reported elsewhere.

**Table 2:** Descriptives and statistical tests for the VAS from the open provocation and double-blind tests for sensitive and control participants by exposure.

	Sham		GSM		UMTS				Sham vs. GSM		Sham vs. UMTS	
Test	Sensitive [M (SE)]^a^	Control [M (SE)]	Sensitive [M (SE)]	Control [M (SE)]	Sensitive [M (SE)]	Control [M (SE)]	Sensitive vs. control (*F*)^b^	Group by condition (*F*)	Sensitive (*t*)	Control (*t*)	Sensitive (*t*)	Control (*t*)
Open provocation												
Anxiety	1.99 (0.26)	1.27 (0.10)	2.47 (0.28)	1.31 (0.11)	2.82 (0.32)	1.33 (0.11)	14.85**	14.30**	4.01**	0.52	5.23**	0.68
Tension	2.05 (0.27)	1.34 (0.11)	2.65 (0.30)	1.35 (0.11)	2.81 (0.31)	1.41 (0.12)	14.59**	15.65**	4.97**	0.52	5.18**	0.60
Arousal	1.96 (0.26)	1.18 (0.10)	2.61 (0.29)	1.19 (0.10)	2.72 (0.30)	1.22 (0.11)	17.26**	20.51**	4.72**	−0.16	6.14**	−0.22
Relaxation^c^	6.69 (0.34)	8.06 (0.15)	6.06 (0.35)	7.98 (0.16)	6.06 (0.36)	8.01 (0.16)	26.16**	7.39**	3.49**	0.97	3.39**	0.14
Discomfort	2.44 (0.29)	1.37 (0.13)	3.21 (0.30)	1.41 (0.12)	3.30 (0.32)	1.39 (0.13)	28.58**	15.38**	4.02**	0.46	4.47**	−0.14
Fatigue	3.26 (0.33)	1.97 (0.16)	3.21 (0.32)	1.91 (0.16)	3.40 (0.33)	2.00 (0.16)	14.52**	0.33				
Double-blind												
Anxiety	2.14 (0.26)	1.82 (0.12)	2.50 (0.27)	1.77 (0.13)	2.82 (0.31)	1.67 (0.11)	7.72*	8.15**	1.90	−0.63	2.89	−2.06
Tension	2.28 (0.27)	1.92 (0.12)	2.59 (0.27)	1.87 (0.13)	3.02 (0.33)	1.81 (0.12)	7.41*	8.36**	1.70	−0.72	2.94	−1.59
Arousal	2.17 (0.26)	1.74 (0.12)	2.59 (0.28)	1.71 (0.12)	2.92 (0.31)	1.65 (0.11)	9.6**	8.52**	2.20	−0.22	3.37**	−0.74
Relaxation	6.58 (0.34)	7.38 (0.15)	6.51 (0.32)	7.50 (0.15)	5.97 (0.40)	7.53 (0.16)	10.78**	6.47**	0.74	−1.14	2.15	−2.10
Discomfort	2.39 (0.31)	1.32 (0.11)	2.41 (0.25)	1.30 (0.11)	2.53 (0.30)	1.25 (0.11)	18.27**	0.94				
Fatigue	3.04 (0.37)	1.94 (0.15)	3.00 (0.33)	1.65 (0.13)	2.88 (0.33)	1.67 (0.13)	16.62**	0.82				

Bonferroni correction for multiple comparisons: open provocation *p* < 0.0025; double-blind *p* < 0.003. Nonparametric statistics was also performed on the untransformed data with virtually the same results (copies of this analysis are available upon request).

a Mean (M) and SE are original untransformed data.

b For the open provocation test, one sensitive participant failed to complete any of the VAS, whereas another did not complete any of the fatigue VAS.

c The relaxation VAS was reversed so that a high score indicates extremely relaxed.

* *p* ≤ 0.01.

** *p* ≤ 0.0025.

**Table 3:** Descriptives and statistical tests for level of arousal by session by group.

Session	Sham [M (SE)]^a^	UMTS [M (SE)]	Condition (*F*)^b^	Group (*F*)	Condition × group (*F*)
Session 2					
Sensitive	2.33 (0.44)	3.52 (0.45)	1.74	8.86*	3.39
Control	1.96 (0.22)	1.69 (0.22)			
Session 3					
Sensitive	2.48 (0.52)	2.66 (0.67)	0.09	3.73	0.26
Control	1.82 (0.23)	1.65 (0.17)			
Session 4					
Sensitive	1.74 (0.37)	2.25 (0.53)	0.73	1.85	0.30
Control	1.39 (0.15)	1.62 (0.19)			

a Mean (M) and SE for original untransformed data.

b Session 2 df = (1,101); session 3 df = (1,104); session 4 df = (1,99).

* *p* ≤ 0.05.

**Table 4:** Medians and Z-scores for total number of symptoms and total symptom score from open provocation and double-blind tests for sensitive and controls by exposure.

	Sham			GSM			UMTS				Sham vs. GSM	Sham vs. UMTS	
Test	Sensitive (median)	Control (median)	Sensitive vs. control (Z)	Sensitive (median)	Control (median)	Sensitive vs. control (Z)	Sensitive (median)	Control (median)	Sensitive vs. control (Z)	Sensitive (Z)	Control (Z)	Sensitive (Z)	Control (Z)
Open provocation													
Total number of symptoms	2.00	0.00	−4.88*	5.00	1.00	−7.65*	5.00	1.00	−7.84*	−3.67*	−1.54	−4.57*	−2.91*
Total symptom score	2.00	0.00	−5.21*	5.50	0.00	−7.88*	6.00	1.00	−8.09*	−3.45*	−1.80	−4.64*	−2.55
Double-blind													
Total number of symptoms	3.00	0.33	−6.86*	3.00	0.33	−6.72*	3.33	0.33	−7.06*	−0.70	−0.05	−1.65	−0.83
Total symptom score	3.33	0.33	−6.33*	4.00	0.33	−6.32*	4.00	0.17	−7.05*	−0.24	−0.69	−1.56	−0.17

Bonferroni corrections: sensitive vs. control *p* = 0.008; sham vs. GSM, sham vs. UMTS *p* = 0.006.

* *p* ≤ 0.005.

**Table 5:** Descriptives and statistical tests for physiological measures for sensitive and control participants by exposure during open provocation and double-blind tests.

	Sham		GSM		UMTS				
Test	Sensitive [M (SE)]^a^	Control [M (SE)]	Sensitive [M (SE)]	Control [M (SE)]	Sensitive [M (SE)]	Control [M (SE)]	Condition (*F*)	Sensitive vs. control (*F*)	Group by condition (*F*)
Open provocation									
BVP M^b^	34.34 (0.06)	34.39 (0.04)	34.30 (0.07)	34.38 (0.04)	34.32 (0.06)	34.38 (0.04)			
BVP SD	2.23 (0.23)	2.51 (0.17)	2.07 (0.22)	2.50 (0.16)	2.17 (0.23)	2.50 (0.17)	1.38	2.89	1.70
SC M	5.36 (0.52)	3.47 (0.21)	5.50 (0.50)	3.46 (0.20)	5.53 (0.51)	3.43 (0.21)	0.32	21.82##	1.22
SC SD	0.62 (0.08)	0.45 (0.03)	0.62 (0.07)	0.45 (0.03)	0.64 (0.07)	0.46 (0.03)	0.70	6.78**	0.51
HR M	67.73 (1.21)	66.27 (0.88)	68.35 (1.27)	66.06 (0.89)	68.82 (1.46)	66.22 (0.89)	1.74	1.44	2.24
HR SD	6.60 (0.56)	5.77 (0.32)	6.18 (0.46)	5.80 (0.33)	6.73 (0.54)	5.76 (0.34)	0.43	5.35*	1.05
Double-blind									
BVP M	34.29 (0.05)	34.34 (0.03)	34.29 (0.10)	34.36 (0.04)	34.40 (0.06)	34.37 (0.04)			
BVP SD	2.52 (0.26)	2.73 (0.15)	2.45 (0.23)	2.67 (0.16)	2.48 (0.24)	2.92 (0.15)	0.78	0.59	0.59
SC M	5.52 (0.54)	3.96 (0.22)	5.39 (0.45)	3.86 (0.23)	6.12 (0.57)	4.34 (0.27)	2.81	15.14##	0.08
SC SD	1.07 (0.13)	0.83 (0.07)	1.14 (0.13)	0.79 (0.06)	1.17 (0.12)	0.88 (0.07)	1.36	8.55#	0.38
HR M	72.80 (1.41)	71.95 (1.03)	73.80 (1.53)	71.55 (0.97)	73.21 (1.46)	71.41 (0.99)	0.23	0.89	0.79
HR SD	7.77 (0.85)	7.18 (0.34)	7.27 (0.62)	7.65 (0.39)	7.75 (0.68)	7.24 (0.35)	0.05	0.01	1.63

Abbreviations: BVP, blood volume pulse; HR, heart rate; M, mean; SC, skin conductance. Nonparametric statistics was also performed on the untransformed data with virtually the same results (copies of this analysis are available upon request).

a Mean and SE for original untransformed data.

b BVP M data did not lend themselves to transformation as participants’ scores were tightly grouped around the mean; therefore, ANOVAs were not conducted on these data.

* *p* ≤ 0.05.

** *p* ≤ 0.01.

# *p* ≤ 0.005.

## *p* ≤ 0.001.
